# Supplementary material for: Splice-Junction-Based Mapping of Alternative Isoforms in the Human Proteome
Source: Cell Rep. Author manuscript; Available in PMC 2020 Jan 15. (PMC6961840; doi:10.1016/j.celrep.2019.11.026)
Supplement: 2 [file NIHMS1546469-supplement-2.zip › DF1/PXD010154/Thyroid/Thyroid_36_SKIV2L_METAATLLRRDIVFAASLYTNR.pdf]

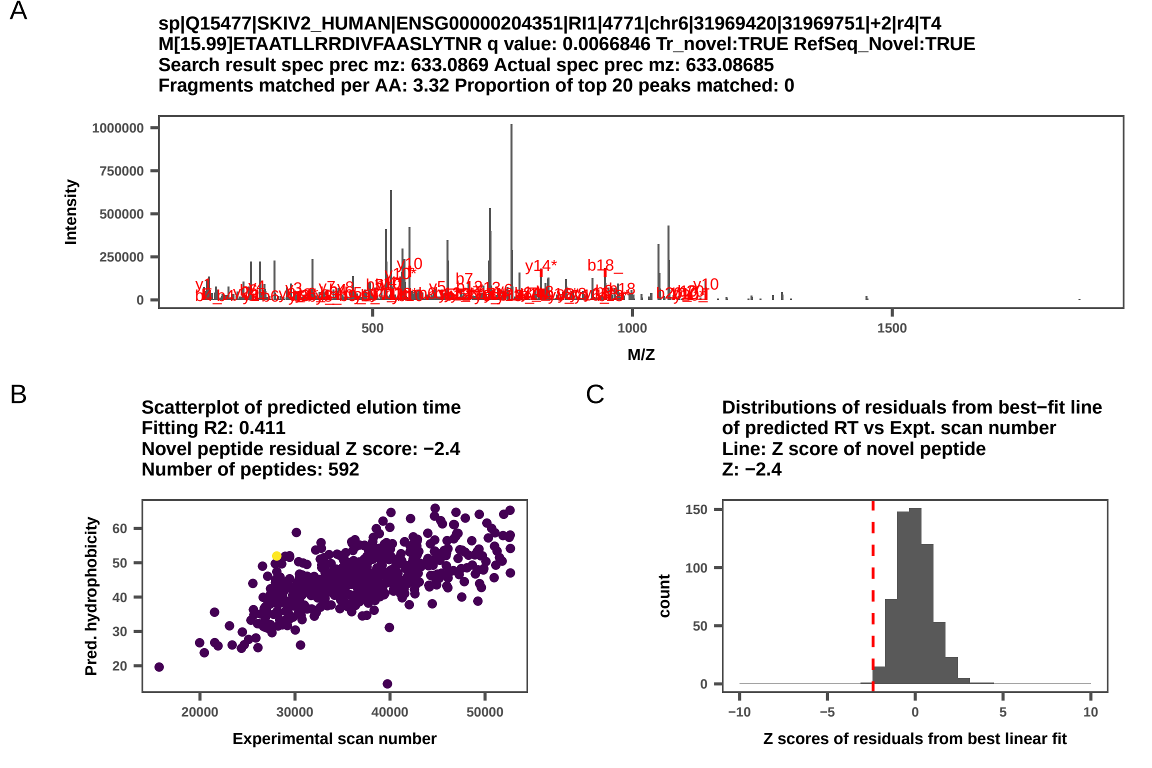

A
sp|Q15477|SKIV2_HUMAN|ENSG00000204351|RI1|4771|chr6|31969420|31969751|+2|r4|T4
M[15.99]ETAATLLRRDIVFAASLYTNR q value: 0.0066846 Tr_novel:TRUE RefSeq_Novel:TRUE
Search result spec prec mz: 633.0869 Actual spec prec mz: 633.08685
Fragments matched per AA: 3.32 Proportion of top 20 peaks matched: 0
Intensity
1000000
750000
500000
250000
0
y10
y14*
b18_
b7
y10
500
1000
1500
M/Z
B
Scatterplot of predicted elution time
Fitting R2: 0.411
Novel peptide residual Z score: −2.4
Number of peptides: 592
Pred. hydrophobicity
60
50
40
30
20
20000
30000
40000
50000
Experimental scan number
C
Distributions of residuals from best−fit line
of predicted RT vs Expt. scan number
Line: Z score of novel peptide
Z: −2.4
count
150
100
50
0
−10
−5
0
5
10
Z scores of residuals from best linear fit
